# Predicting Inflammatory Bowel Disease Flares Using Artificial Intelligence and Remote Monitoring: Toward Proactive Disease Management

**DOI:** 10.7759/cureus.107770

**Published:** 2026-04-26

**Authors:** Mohammad Alali, Mouhammad Alyounes, Mohammad Alkhaleel alomar

**Affiliations:** 1 Internal Medicine, Henry Ford Hospital, Macomb, USA; 2 General Medicine, Alexandria Faculty of Medicine, Alexandria, EGY

**Keywords:** artificial intelligence, flare prediction, inflammatory bowel disease, machine learning, telemedicine

## Abstract

Inflammatory bowel disease (IBD), including Crohn’s disease and ulcerative colitis, is characterized by a chronic relapsing-remitting course with unpredictable disease flares that significantly contribute to morbidity, impaired quality of life, and increased healthcare utilization. Despite advances in biologic therapies and treat-to-target strategies, disease management remains largely reactive, relying on clinical symptoms and biomarkers that often reflect established inflammation rather than anticipate disease exacerbation.

Recent advances in artificial intelligence (AI) and remote monitoring technologies offer a paradigm shift toward proactive disease management. Machine learning models can integrate and analyze complex, multidimensional datasets - including clinical, biochemical, and behavioral data - to identify patterns predictive of disease flares. Concurrently, telemedicine platforms and digital health tools enable continuous collection of patient-generated health data, facilitating real-time disease monitoring.

This narrative review synthesizes current evidence on AI-based predictive modeling in IBD and evaluates the role of remote monitoring tools, including patient-reported outcomes, biomarkers, and telemedicine platforms. It further explores the integration of these approaches into a unified predictive framework aimed at early identification of disease flares and timely clinical intervention. While emerging data demonstrate promising predictive performance, challenges related to validation, generalizability, and clinical implementation remain significant.

## Introduction and background

Inflammatory bowel disease (IBD) is a chronic inflammatory disorder of the gastrointestinal tract characterized by periods of remission and relapse. Disease flares represent acute exacerbations of inflammation and are associated with increased hospitalization rates, corticosteroid exposure, and long-term complications, including strictures and fistula formation [1].

Despite significant therapeutic advancements, including biologic and small-molecule agents, the management of IBD remains largely reactive. Clinical decision-making is typically based on symptoms, laboratory markers, and endoscopic findings, which often reflect active inflammation rather than predict future disease activity [2,3]. This reactive approach contributes to delayed intervention and suboptimal disease control.

Biomarkers such as fecal calprotectin and C-reactive protein (CRP) have improved disease monitoring; however, their predictive accuracy for future flares is variable and influenced by disease phenotype and patient-specific factors [4-6]. As a result, there is an unmet need for predictive tools capable of identifying patients at risk for disease exacerbation prior to clinical deterioration.

Artificial intelligence (AI), particularly machine learning, has emerged as a powerful tool for predictive analytics in healthcare. By leveraging large datasets and identifying complex nonlinear relationships, AI models offer the potential to predict disease activity and treatment response in IBD [7]. Simultaneously, the adoption of remote monitoring technologies - including telemedicine platforms, mobile applications, and wearable devices - has enabled continuous, real-time data collection outside traditional clinical settings [8-10].

These predictive models typically integrate multimodal data, including clinical variables, biomarkers, patient-reported outcomes, and wearable-derived physiologic data. Commonly utilized machine learning approaches include regression-based models, random forest algorithms, gradient boosting methods, and deep learning techniques, each offering unique advantages in handling complex and high-dimensional datasets.

The convergence of AI-driven analytics with remote monitoring systems represents a novel opportunity to transition IBD management from a reactive to a proactive model. This review examines current evidence supporting this approach and is novel in its focus on integrating artificial intelligence with remote monitoring technologies into a unified predictive framework aimed at enabling proactive, rather than reactive, disease management.

## Review

Literature search strategy

This narrative review was conducted through a comprehensive literature search of electronic databases, including PubMed and Google Scholar. Keywords used included “inflammatory bowel disease,” “machine learning,” “artificial intelligence,” “remote monitoring,” “telemedicine,” and “flare prediction.” Relevant articles published between 2015 and 2026 were reviewed. Studies were selected based on relevance, methodological quality, and applicability to predictive modeling and remote monitoring in IBD. Additional references were identified through manual review of cited literature.

Clinical burden and definition of IBD flares

IBD flares are typically defined by a combination of worsening clinical symptoms, biochemical markers, and endoscopic findings, although standardized definitions remain inconsistent across studies [1]. The heterogeneity in flare definitions complicates both clinical management and research efforts.

Flares are associated with significant clinical and economic burden, including increased healthcare utilization, reduced quality of life, and risk of disease progression [2]. Furthermore, recurrent flares contribute to cumulative bowel damage and disability over time.

The Selecting Therapeutic Targets in Inflammatory Bowel Disease (STRIDE-II) consensus emphasizes the importance of achieving sustained remission and mucosal healing as therapeutic targets [3]. However, the inability to accurately predict disease flares limits the effectiveness of treat-to-target strategies and underscores the need for predictive tools.

Artificial intelligence in IBD: predictive applications

Artificial intelligence has been increasingly utilized in IBD for predictive modeling across multiple domains, including disease activity, therapeutic response, and long-term outcomes [7].

Machine learning algorithms have demonstrated the ability to predict disease activity using clinical and laboratory data. Cai et al. reported that machine learning models can effectively classify disease activity with high accuracy, with reported performance metrics demonstrating strong predictive accuracy and discrimination [11]. Similarly, Zand et al. demonstrated that predictive analytics can identify patients at risk for adverse outcomes, highlighting the ability of machine learning models to utilize routinely collected clinical data for risk stratification and early intervention [12]. Commonly used machine learning approaches in this context include random forest, gradient boosting, and deep learning models, which are particularly well-suited for analyzing complex and longitudinal data, including time-series data derived from wearable devices.

AI has also been applied to predict therapeutic response, an area of significant clinical importance. Park et al. developed a machine learning model capable of predicting non-durable response to anti-TNF (tumor necrosis factor) therapy, while He et al. utilized gene expression-based approaches to predict response to biologic agents [13,14]. These models have the potential to support personalized treatment strategies and reduce trial-and-error prescribing.

In addition, predictive models have been developed to estimate remission and relapse risk. Park et al. demonstrated the feasibility of clinical decision support tools in predicting remission and relapse in Crohn’s disease patients [15]. More advanced applications incorporate imaging and histologic data. Stidham et al. utilized AI to quantify cumulative disease severity using imaging modalities, while Iacucci et al. demonstrated that histology-based AI models can predict clinical outcomes and disease progression [16,17].

Despite these promising developments, limitations remain, including small sample sizes, heterogeneity in datasets, lack of external validation, and challenges in model interpretability. 

Remote monitoring in IBD

Remote monitoring has emerged as a critical component of modern IBD care, enabling continuous assessment of disease activity and facilitating early intervention.

The myIBDcoach randomized controlled trial demonstrated that telemedicine-based management improved disease monitoring, reduced outpatient visits, and maintained disease control, supporting the feasibility of remote care models [18].

Patient-reported outcomes (PROs) play a central role in remote monitoring by providing real-time insights into symptom burden and functional status. PRO-based tools have been validated as reliable indicators of disease activity and are increasingly incorporated into digital health platforms [9,19].

Biomarkers remain essential in monitoring disease activity. Fecal calprotectin is a sensitive marker of intestinal inflammation and has been shown to predict relapse, making it particularly valuable for remote monitoring strategies [4,20]. CRP and other biomarkers complement clinical assessment but are less specific and may be influenced by systemic inflammation [5,6].

Together, these tools generate high-frequency, longitudinal datasets that provide a comprehensive view of disease activity and are well-suited for integration with AI-based predictive models.

Integration of AI and remote monitoring: a proactive care model

**Figure 1:**
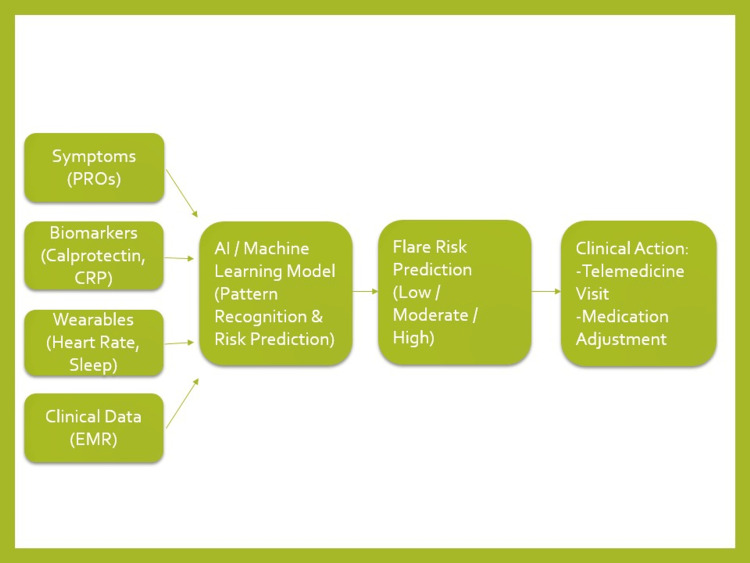
AI-Driven Prediction of IBD Flares Using Remote Monitoring Data Integration of artificial intelligence and remote monitoring for prediction of inflammatory bowel disease flares. Patient-generated data, including symptoms, biomarkers (fecal calprotectin, C-reactive protein), wearable metrics, and clinical data, are analyzed using machine learning models to generate individualized flare risk predictions, enabling timely clinical intervention and proactive disease management. Image credit: This figure was created using Microsoft PowerPoint (Microsoft, Redmond, WA) by Dr. Mohammad Alali.

The integration of AI with remote monitoring represents a transformative approach to IBD management, enabling continuous risk assessment and early intervention. A conceptual framework illustrating this integration is shown in Figure 1.

In this model, patient-generated data - including symptoms, biomarkers, and behavioral metrics - are continuously collected through digital platforms. Machine learning algorithms analyze these multidimensional datasets to identify patterns associated with disease exacerbation and generate individualized risk predictions.

These predictions can be used to trigger timely clinical interventions, such as medication adjustments, additional diagnostic testing, or telemedicine consultations. This approach has the potential to reduce hospitalizations, prevent disease progression, and improve overall patient outcomes.

Recent studies support the feasibility of this approach. A 2026 study demonstrated that machine learning models incorporating clinical, lifestyle, and psychosocial data from remote monitoring platforms could predict IBD flares with moderate accuracy (area under the curve (AUC) ~0.77) [21], highlighting the potential for early identification of high-risk patients and enabling timely intervention to prevent disease progression and reduce healthcare utilization.

The Transparent Reporting of a Multivariable Prediction Model for Individual Prognosis or Diagnosis-Artificial Intelligence (TRIPOD-AI) framework emphasizes the importance of transparency, reproducibility, and validation in predictive modeling, which will be essential for translating these tools into clinical practice [22].

Challenges and limitations

Despite promising advances, several challenges limit the widespread adoption of AI-driven predictive models in IBD. Most studies remain retrospective and lack external validation, which limits generalizability across diverse patient populations [23]. In addition, integration of predictive models into clinical workflows and electronic health records remains technically complex.

Data privacy and security concerns are also significant, particularly given the continuous collection of sensitive patient data. Algorithmic bias and limited interpretability may further reduce clinician trust and hinder adoption.

In addition, comprehensive evaluation of predictive models - including calibration, discrimination, and clinical utility - is often lacking, highlighting the need for standardized methodologies [24].

Additional barriers to implementation include cost-effectiveness, patient adherence, and regulatory considerations. The integration of artificial intelligence and remote monitoring platforms may require substantial initial investment in digital infrastructure, data integration systems, and clinician training, raising concerns about cost-effectiveness, particularly in resource-limited settings.

Patient adherence represents another critical factor, as the effectiveness of remote monitoring depends on consistent engagement with digital tools, including regular symptom reporting, biomarker testing, and wearable device use. Variability in adherence may impact data quality and the reliability of predictive models.

Furthermore, regulatory and ethical challenges remain significant. The use of AI in clinical decision-making raises concerns regarding data privacy, security, algorithm transparency, and accountability. Regulatory frameworks for AI-based medical tools are still evolving, which may limit widespread clinical adoption.

Future directions

Future research should focus on developing robust, externally validated predictive models that integrate diverse data sources, including biomarkers, PROs, and wearable-derived metrics.

Advances in wearable technology and digital health platforms may enable continuous physiologic monitoring, further enhancing predictive accuracy. Integration with telemedicine systems could facilitate real-time clinical decision-making and personalized treatment strategies.

Collaborative efforts between clinicians, data scientists, and healthcare systems will be essential to overcome existing barriers and translate these innovations into routine clinical practice.

## Conclusions

AI and remote monitoring technologies offer a promising pathway toward proactive management of IBD. By enabling early prediction of disease flares, these approaches have the potential to improve patient outcomes and reduce healthcare burden. However, further research is required to validate predictive models, address real-world implementation challenges - including cost-effectiveness, patient adherence, and regulatory considerations - and ensure their effective integration into clinical practice.
